# Developmental regulation of histone H2A deposition via serine-1 phosphorylation

**DOI:** 10.1186/1756-8935-6-S1-P74

**Published:** 2013-03-18

**Authors:** Wei-Lin Wang, Joshua J Nicklay, Lissa Anderson, Joe Strukl, Takashi Onikubo, Jeffrey Shabanowitz, Donald F Hunt, David Shechter

**Affiliations:** 1 Department of Biochemistry, Albert Einstein College of Medicine, Yeshiva University, Bronx, NY, USA; 2 Department of Chemistry, University of Virginia, Charlottesville, VA, USA

## 

H2A/H4 serine 1 phosphoryation (S1ph) is hypothesized to participate in many aspects of chromatin metabolism, including DNA replication, repair, recombination, mitosis, and regulation of gene expression. However, few specific functions for this ubiquitous histone modification have been demonstrated. In early *Xenopus laevis* embryos, development is tightly regulated to prevent premature zygotic transcription post-fertilization and to activate zygotic gene expression in a timely manner simultaneous with the mid-blastula transition (MBT). Rapid histone deposition occurs in *Xenopus* embryos and in sperm pronuclei assembled in cell-free *Xenopus* egg extract, concomitant with sperm decondensation and DNA replication. *In vivo* we observed that H2AS1ph appeared upon oocyte germinal vesicle breakdown (GVBD) while H4S1ph only appeared post-MBT. This is the first study to demonstrate a biological distinction between H2A and H4 S1 phosphorylation. H2A S1ph abundance in chromatin was not dependent upon DNA replication nor did it exhibit any cell-cycle changes in chromatin in cycling cell-free extract. However H4S1ph was observed on pronuclei assembled in cell-free extract in a DNA concentration-dependent fashion. The appearance of H4S1P highly correlates with the increased nuclear-to-cytoplasmic ratio found at the beginning of maternal gene expression at the MBT. Addition of wildtype H2A (H2Awt), H2AS1A (unphosphorylatable), and H2AS1E (phosphomimetic) mutant histone H2A/H2B dimers to cell-free extract showed that H2AS1A had a slower rate of chromatin incorporation than either H2Awt or H2AS1E. In cell-free extract H2Awt and H2AS1E, but not the unphosphorylated H2AS1A, interact with the histone storage chaperone Nucleoplasmin. All three interact with Nap1. Finally, overexpression of H2AS1A in Xenopusembryos results in more than 50% embryonic lethality. We conclude that H2A serine 1 phosphorylation is necessary for proper entrance into the histone chaperone pathway in the early embryo.

